# Immune and non-immune mediators in the fibrosis pathogenesis of salivary gland in Sjögren’s syndrome

**DOI:** 10.3389/fimmu.2024.1421436

**Published:** 2024-10-14

**Authors:** Danbao Ma, Yun Feng, Xiang Lin

**Affiliations:** ^1^ School of Chinese Medicine, The University of Hong Kong, Hong Kong, Hong Kong SAR, China; ^2^ Department of Ophthalmology, Peking University Third Hospital, Beijing, China; ^3^ Department of Chinese Medicine, the University of Hong Kong-Shenzhen Hospital (HKU-SZH), Shenzhen, China

**Keywords:** Sjögren’s syndrome, Sjögren’s disease (SjD), salivary gland, fibrosis, infection, epithelial-mesenchymal transition, extracellular matrix remodeling

## Abstract

Sjögren’s syndrome (SS) or Sjögren’s disease (SjD) is a systemic autoimmune disease clinically manifested as sicca symptoms. This disease primarily impacts the functionality of exocrine glands, specifically the lacrimal and salivary glands (SG). SG fibrosis, an irreversible morphological change, is a severe consequence that occurs in the later stages of the disease due to sustained inflammation. However, the mechanism underlying SG fibrosis in SS remains under-investigated. Glandular fibrosis may arise from chronic sialadenitis, in which the interactions between infiltrating lymphocytes and epithelial cells potentially contributes to fibrotic pathogenesis. Thus, both immune and non-immune cells are closely involved in this process, while their interplays are not fully understood. The molecular mechanism of tissue fibrosis is partly associated with an imbalance of immune responses, in which the transforming growth factor-beta (TGF-β)-dependent epithelial-mesenchymal transition (EMT) and extracellular matrix remodeling are recently investigated. In addition, viral infection has been implicated in the pathogenesis of SS. Viral-specific innate immune response could exacerbate the autoimmune progression, resulting in overt inflammation in SG. Notably, post-COVID patients exhibit typical SS symptoms and severe inflammatory sialadenitis, which are positively correlated with SG damage. In this review, we discuss the immune and non-immune risk factors in SG fibrosis and summarize the evidence to understand the mechanisms upon autoimmune progression in SS.

## 1 Introduction

Sjögren’s syndrome (SS) or Sjögren’s disease (SjD) is an autoimmune disease that primarily causes dryness of the exocrine glands, in which the salivary and lacrimal glands are predominantly affected. Consistent with other autoimmune diseases, SS displays an unbalanced gender ratio, with a male-to-female ratio of approximating 1:20 according to the latest research, and predominantly occurs in middle-aged women (1). Primary SS (pSS) is defined as the occurrence of SS independently, without the concurrent presence of other rheumatic diseases (2). In contrast, secondary SS is consistently associated with other autoimmune complications, such as rheumatoid arthritis (RA), and systemic lupus erythematosus (SLE), and multiple sclerosis. The disease typically manifests with sicca symptoms, including reduced salivary volume, and systemic manifestations including fatigue, joint, and muscle pain. In later stages, SS can progress to B-cell lymphoma, which is one of the leading causes of disease-associated mortality. Other mortality risks include older age, male gender, vasculitis, cryoglobulinemia and interstitial lung disease (ILD) (3). SS was initially recognized as autoimmune sialadenitis, due to the characteristic infiltration of massive lymphocytes in the salivary gland (SG) and the subsequent development of localized chronic inflammation. Immune activation in the epithelium and the accumulation of lymphocytic infiltrates result in the formation of focal lymphocytic sialadenitis (FLS) within the SG. The identification of Sjögren’s lymphocytic sialadenitis serves as the gold standard for the assessment of SS (4). In patients with SS, aberrant T cell and B cell responses to autoantigens not only lead to the overproduction of inflammatory cytokines and pathogenic autoantibodies but also drive chronic inflammation in the epithelium of the exocrine glands. Furthermore, prolonged inflammation may result in irreversible damage or even more severe organ damage to the SG, such as atrophy and fibrosis.

Fibrosis is a common feature of connective tissue diseases associated with autoimmune disorders. Tissue fibrosis represents a remodeling process of the extracellular matrix in response to injury from acute or chronic stimulation. This involves the replacement of healthy parenchymal tissue with fibrous connective tissue. In the absence of disease, the accumulation of collagen fibers leads to tissue hardening and scarring. However, excessive deposition can initiate the loss of tissue architecture, which may ultimately result in organ dysfunction and failure (5). Non-specific chronic sialadenitis (NSCS) may result in pathological fibrosis in the SG of patients with SS and is likely to be associated with focal lymphocyte infiltration (6). The prevailing hypothesis is that chronic inflammation is the primary driver of local damage and the progressive fibrotic transformation of tissue. Fibrosis has been investigated in several organs in patients with SS, including the lungs, livers, and kidneys, but less is understood about fibrosis in the SG. In fact, this organic lesion results in permanent destruction of the SG, causing significant distress to the patient and necessitating appropriate attention. This pathological feature tends to worsen with age and is difficult to reverse, thus severely impacting the quality of life for the elderly with dental damage and reduced salivary flow (7–9). Early evidence has documented the age-related fibrosis of the SG in patients with SS (10).

Indeed, age is associated with reduced acini, ductal dilation, fatty infiltration and fibrosis (11), but it is not the only factor that contributes to SG fibrosis. As a common consequence of tissue injury and inflammation, fibrosis has been found to be positively correlated with the focus score in minor SG of patients with SS (12). Consequently, the presence of focal lymphocytes plays an important role in the context of fibrosis. Pro-inflammatory cytokines and chemokines, including type I interferon (IFN), tumor necrosis factor alpha (TNFα), transforming growth factor-beta (TGF-β), C-X-C motif chemokine ligand 10 (CXCL10), CXCL13, chemokine (C-C motif) ligand 19 (CCL19) and CCL21, are highly expressed in SGs and are responsible for recruiting immune cells. Focal accumulations of lymphocytes, macrophages and plasma cells typically form fibrosis-related ectopic lymphoid structures (ELS) in SG, supporting the activity of autoreactive plasma cells and the persistent inflammatory stimulation in SGs. Collectively, these immune infiltrates contribute to the fibrotic pathology.

The pathogenicity of immune cells, either directly or indirectly involved in SG fibrosis, remains incompletely understood, while recent evidence has begun to shed light on the contributions of various immune cell populations to SG fibrosis. While chronic inflammation in SS does not inevitably result in fibrosis, once this morphological change has occurred, it is irreversible. Currently, there is no effective therapeutic approach available, largely due to a lack of acknowledgement of the underlying fibrotic mechanisms. This review will discuss the pro-fibrotic role of both immune and non-immune cells, as well as the underlying mechanisms of SG fibrosis in the context of autoimmune progression and infection.

## 2 The pro-fibrotic role of immune cells in salivary gland of pSS

### 2.1 T cells

**Figure 1:**
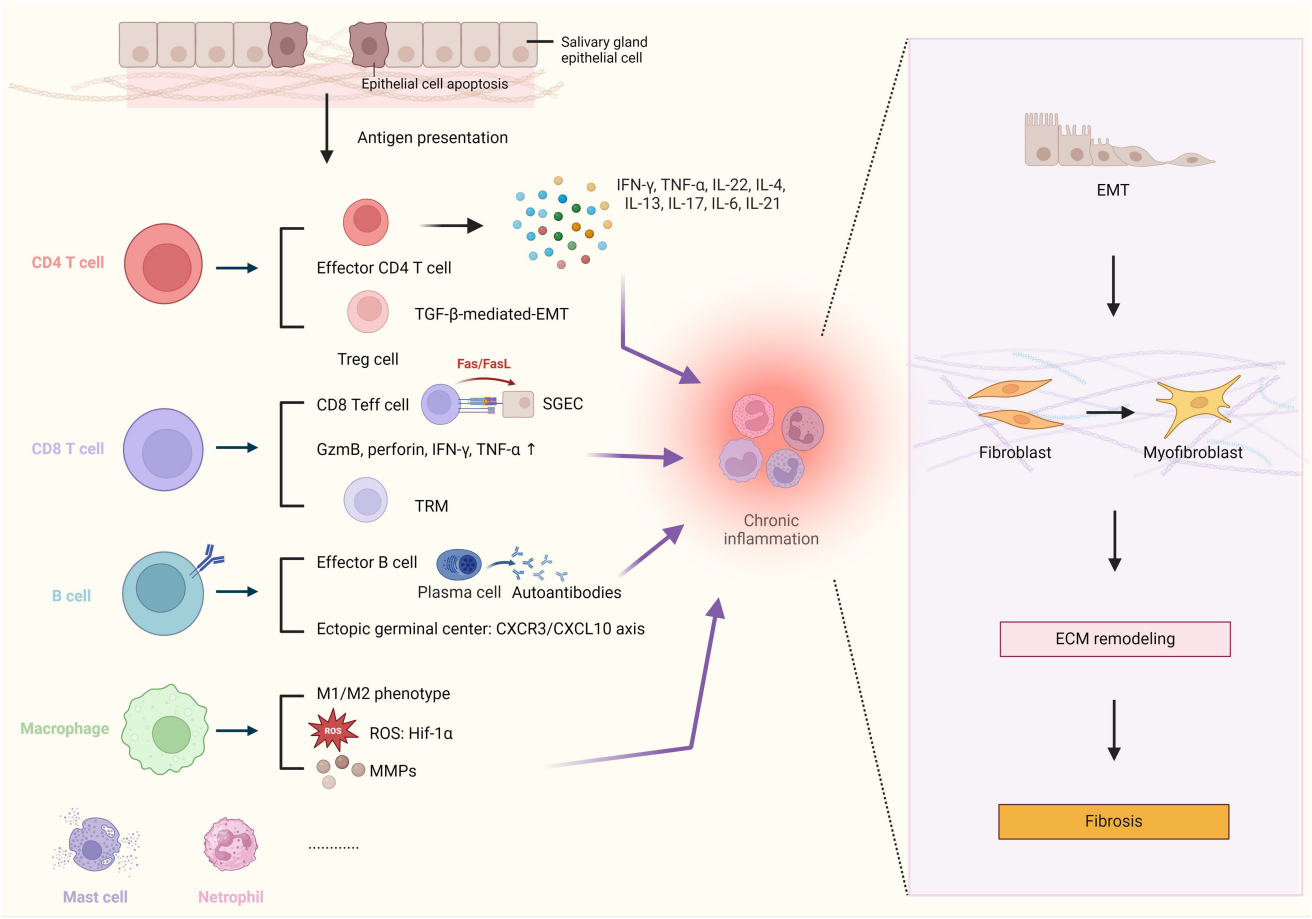
Schematic Illustration of Immune Mediators Involved in Modulation of Fibrosis Pathogenesis in Salivary Glands. Chronic inflammation, caused by various types of immune cells, plays a crucial role in the process of salivary gland (SG) fibrosis, driving the epithelial-mesenchymal transition (EMT) program, myofibroblast differentiation, and extracellular matrix (ECM) remodeling. In patients with Sjögren’s Syndrome, the salivary epithelium is often damaged, and numerous epithelial cells undergo apoptosis. The autoimmune reaction *in situ* is initiated by autoantigens released by apoptotic salivary gland epithelial cells (SGECs). The chronic inflammation in the SG is primarily caused by pro-inflammatory cytokines from effector T cells, such as interferon-gamma (IFN-γ), tumor necrosis factor-alpha (TNF-α), interleukin-21 (IL-21), and interleukin-6 (IL-6). Transforming growth factor-beta (TGF-β), secreted by regulatory T cells (Treg cells), is the key regulator of EMT-driven fibrosis. CD8^+^ T cells can exacerbate tissue damage by inducing SGECs apoptosis in contact manner, via the Fas/Fas ligand (FasL) pathway and other effector molecules e.g granzyme B, perforin, IFN-γ, and TNF-α. B cells could differentiate into plasma cells and participate in the formation of ectopic germinal centers via the C-X-C motif chemokine receptor 3/chemokine ligand 10 (CXCR3/CXCL10) axis. Macrophages contribute to fibrosis by producing matrix metalloproteinases (MMPs), associated with enhanced the expression of the reactive oxygen species (ROS)-related gene hypoxia-inducible factor 1-alpha (Hif-1α). Created by BioRender.com.

A variety of immune cells are observed to infiltrate the SG, including CD4^+^ T cells, CD8^+^ T cells, B cells, macrophages, dendritic cells (DCs), and mast cells. Additionally, non-immune cells such as fibroblasts are also present. Self-reactive immune cells respond to glandular-specific antigens, resulting in the onset of inflammation and subsequent damage to the SG, which ultimately leads to the development of fibrosis (**Figure 1**). In the initial stages of fibrosis, there is an observable expansion of T cells, particularly CD4^+^ T cells, in dysfunctional glands. Upon examination of histopathological specimens of SG, it was found that clonally expanded CD4^+^ T cells from memory T cells were found to be positively correlated with the grade of fibrotic damage (13). A single-cell analysis approach was employed by researchers, who analyzed data and discovered that the proliferation of CD4^+^ T cells in inflammatory lesions was specifically driven by SG autoantigens from unrelated SS individuals. Moreover, two distinct functional T cell receptor α (TCRα) chains were identified in these hyperactive SG CD4^+^ T cells, in contrast to those observed in peripheral blood of patients with SS. Given that dual TCR-expressing cells are typically present in autoimmune diseases (14), the enrichment of overactive CD4^+^ T cells implicates a heightened autoimmune response in the SG and increased severity of SS. Further investigation is warranted into the role of CD4^+^ T cell subsets in SG fibrosis. It is noteworthy that the majority of local resident T cells within inflammatory site express the CD45RO memory marker, as well as effector hallmarks such as the IL-2 receptor (15). The activation of memory T cells and the proliferation of autoreactive CD4^+^ T cells together indicate that the process is antigen-driven *in situ*. Indeed, the abundance of antigen-experienced CD4^+^CD45RA^−^ T cells have been demonstrated to have a positive correlation with the levels of serum autoantibodies Ro, biopsy focus score and the SG fibrosis (16). The study analyzed the gene expression profile of these CD4^+^CD45RA^-^ memory T cells and characterized their distinct T follicular helper (Tfh) signature in the SG of pSS cases, using two independent gene set enrichment analysis (GSEA) approaches. Notably, not only do CD4^+^ memory T cells play a role, but the absolute number of CD8^+^ memory T cells also correlates with fibrotic morphology, suggesting the complexity of infiltrates in inflamed salivary tissues.

#### 2.1.1 Th1 cells

The exact involvement of Th1 cells in the pathogenesis of SG fibrosis is not well understood, as there have been only a limited number of studies addressing this issue. Th1 cells represent the predominant population responsible for secreting IFN-γ and IL-2. IFN-γ functions as an anti-fibrotic cytokine in idiopathic pulmonary fibrosis (IPF) (17). The prevalence of Th2-type response over Th1 may be indicative of the severity of IPF. In general, Th1 cells play an anti-fibrotic role in organ fibrosis, including lung, kidney, and liver (18). However, this subset may also exhibit opposite functions in SS, although downregulation of IFN-γ can promote the occurrence of autoimmune diseases (19–21). Elevated Th1 population versus Th2 has been observed in SS (22, 23). In contrast to anti-fibrotic effects of IFN-γ, it has been proposed that a distinct Th1 signature is associated with the severity of SS (24). The imbalance of Th1/Th2 in the pathogenesis of SS has long garnered interest. Th1 cells have been shown to highly express STAT-4 and T-bet, in the local SG of SS patients compared to non-SS individuals (25). Th1-associated IFN-γ act as anti-fibrotic agents due to their inhibitory effect on fibroblast and collagen accumulation. Conversely, they induce proinflammatory macrophages and promote local inflammation in the SG (26, 27). Interactions with these macrophages further give rise to Th1 cell response and the production of additional pro-inflammatory cytokines, which may indirectly contribute to chronic inflammation and SG fibrosis. The overproduction of IFN-γ promotes CXCL10 expression, which in turn facilitates the recruitment of immune cells to the site of inflammation by binding to the receptor CXCR3 (28, 29). CXCL10 and other CXCR3 ligands can attract B cells into the inflamed epithelium (30, 31). The accumulation of inflamed tissue might progress into fibrosis in SG.

IFN-γ is able to activate JAK1/2-induced phosphorylation of STAT1 by binding to IFN-γ receptor-1 and -2 (32). It is interesting that IFN-γ can induce ferroptosis of salivary gland epithelial cells (SGECs) via JAK/STAT1 signaling and contribute to the pathogenicity of SS (33). Inhibition of IFN-γ signaling by using the IFN-γ monoclonal antibody (XMG1.2) in non-obese diabetic (NOD) mice can induce glutathione (GSH) and glutathione peroxidase 4 (GPX4), and relieve the SGEC ferroptosis and progression of SS (33). This provides evidence for the controversial functions of IFN-γ in the pathogenesis of SS. The large amount of the programmed death of SGEC such as ferroptosis may ultimately lead to SG fibrosis.

#### 2.1.2 Th2 cells

Active Th2 transcripts predominantly appear in the later stages of SS (34). A distinctive Th2 profile is associated with the formation of germinal centers and marked B cell infiltration in the SG (24). The activation of GATA-3 and STAT-6 regulates the polarization of naive T cells into the Th2 phenotype, subsequently initiating the production of Th2 cytokines, including IL-4, IL-5, IL-10, and IL-13 (23). Some of these cytokines are capable of inducing fibrosis in tissues (35, 36). The role of type 2 response-dependent fibrosis in tissue repair has been recently elucidated (36). Pathogens, including parasites, helminths, and allergens, can provoke type 2 inflammation to support tissue repair and regeneration (36). However, excessive type 2 immunity may lead to the development of allergic diseases and pathological fibrosis (36, 37). Type 2 cytokines indirectly contribute to fibrotic pathology by interacting with associated immune cells. IL-4 and IL-13 activate tissue-repair macrophages and initiate tissue regeneration (36). These tissue-resident macrophages play a pivotal role in pathological fibrosis following injury, as they secrete cytokines such as TGF-β1, which induce the differentiation of fibroblasts into myofibroblasts (37). The activation of myofibroblast further facilitates the synthesis of extracellular matrix (ECM) (38). Local fibroblasts may be influenced by tissue-specific macrophages in the context of a chronic infection in SG, with the mediation of the fibrotic cascade occurring under the regulation of Th2 cytokines (36, 37, 39, 40). Additionally, fibroblasts can also respond directly to IL-4 and IL-13 signaling, which stimulates their transformation into myofibroblasts (36, 41). This process consequently increases the deposition of collagen and α-smooth muscle actin (α-SMA), thereby supporting wound healing and subsequent fibrosis (36). In recent studies, it has been demonstrated that IL-13 and IL-22 act in concert to prime and stimulate fibroblasts (42). IL-22 originates from a variety of sources, including lymphocytes and non-lymphocytes (43). However, its production by Th2 cells is rarely documented. Both IL-13 and IL-22 facilitate the proliferation of SG-infiltrated fibroblasts, thereby promoting the establishment of tertiary lymphoid structures (TLS) and the expansion of the fibroblastic network (43–47). Notably, the early priming of resident fibroblasts is regulated by IL-13. Together, type 2 cytokines are involved in SG fibrosis through multiple mechanisms.

#### 2.1.3 Treg cells

Treg cells, a subset of T cells, play an essential role in maintaining immune tolerance. They are of great importance for regulating T cell activity and maintaining immune homeostasis as well as preventing autoimmune responses (48). The differentiation of naïve CD4 T cells into Treg cells requires IL-2 and TGF-β (49). Conversely, TGF-β could be produced by Treg cells. Although TGF-β signaling is indispensable for immunosuppression, it also functions in various other physiological processes. Treg-derived TGF-β, particularly TGF-β1, is a key pro-fibrotic cytokine and serves as the primary driver of epithelial-mesenchymal transition (EMT)-dependent fibrosis in most chronic inflammatory diseases (50) (**Figure 1**). The phenomenon of inflammatory EMT has been observed during the process of wound healing and pathological fibrosis of the SG (51). In patients with primary SS, TGF-β1 contributes to SG fibrosis via the canonical TGF-β1/SMAD/Snail signaling pathway (52). Snail is a downstream transcriptional factor of phosphorylated SMAD 2/3 that promotes the expression of genes associated with EMT. In the context of an inflamed SG (52), TGF-β1 primarily originates from local SGECs, Treg cells, and macrophages. In TGF-β1-exposed SGECs, intracellular signaling cascades result in SMAD 2/3 phosphorylation and further induce EMT-related gene expression, simultaneously leading to the activation of myofibroblasts and ECM reshaping (53). The expression of epithelial markers, such as E-cadherin, on SGECs is down-regulated, while the expression of mesenchymal markers, such as vimentin and type I collagen, is significantly increased. SG inflammation is positively correlated with the acquisition of mesenchymal properties (51). TGF-β1-treated healthy SGECs showed altered morphology with loosened cell-cell adhesion and an elongated shape (54). The effect of TGF-β1 signaling in SG fibrosis may be manifested by the transition of SGECs into mesenchymal cells. These resident mesenchymal cells specifically exhibit a myofibroblast-like phenotype that is associated with progressive SG fibrosis.

Given that TGF-β1 is a vital regulator in SG fibrosis, local targeting TGF-β1 may offer a promising avenue for the treatment of SG fibrosis. The TGF-β1 inhibitor SB-431542 demonstrated significant inhibitory effects on the expression of fibrosis phenotypic markers vimentin and type I collagen in SGECs (52). It has been revealed that the level of SG inflammation is highly connected to EMT-dependent fibrosis (54–56). With regard to the TGF-β-mediated EMT pathway, several studies have mentioned that the pro-inflammatory cytokines IL-6, IL-17, and IL-22, which share this pathway, are also involved in the fibrotic mechanism of SG (53, 55, 57). In addition, TGF-β/IL-17-induced EMT can trigger the fibrotic program of SG via the canonical SMAD pathway or the non-canonical ERK-mediated pathway (53). During EMT, IL-17 stimulates the phosphorylation of SMAD 2/3 and Erk1/2 in SGECs (53). The subsequent section will discuss the functions of IL-17 in greater depth. Nevertheless, the impact of the imbalance between Treg cells and Th17 cells on the SG microenvironment and its role in fibrotic pathogenesis is necessary to be further explored. In the presence of TGF-β and the absence of IL-6, the Treg phenotype is more encouraged than the Th17 phenotype (58). However, it is possible for Treg cells to transdifferentiate into Th17 cells in the presence of pro-inflammatory factors (59). Intriguingly, upon IL-2 stimulation, there is a lower frequency of Treg cells with STAT5 phosphorylation in the peripheral blood of patients with SS (60). STAT5 is a signal transducer that modulates the stabilization of Treg cells. The malfunction and imbalance of Treg cells in SS may be correlated with the severity of the disease. Collectively, Treg cells have the capacity to suppress the development of autoimmune disease as immune suppressors, yet they also produce TGF-β to support fibrotic pathology. Controversially to decreased Treg numbers in autoimmune mouse models, the elevated levels of TGF-β in the local SG indicate the cellular source of TGF-β other than Treg cells, including SGECs and macrophages. However, a study has reported transcriptomic analysis in SG of patients with pSS, which may enhance the Treg profile with a reduced Th17 signature (16). Therefore, the complex interplay between resident Treg cells and SGECs in the context of autoimmune disorders requires further investigation to elucidate, with a particular focus on SG fibrosis.

#### 2.1.4 Th17 cells

Extensive investigation has revealed a correlation between Th17 cells and autoimmune disorders (61, 62). In the combination of IL-6 or IL-21, TGF-β derived from DCs serves as a trigger for Th17 differentiation (63, 64). Indeed, local expression of IL-6, IL-17, and IL-23 increases with the severity of inflammatory lesions in the minor SG of pSS patients (65). Pro-inflammatory IL-17A is the signature cytokine secreted by Th17 cells. Although IL-17 is important in the immune response to extracellular pathogens in mucosal tissues, it induces the production of other inflammatory cytokines and related chemokines that critically contribute to the pathogenesis of autoimmune diseases including pSS. Increased IL-17 mRNA levels have been detected in the SG of patients with SS, concomitant with a reduction in saliva flow rate (65). An augmentation of salivary IL-17 was also detected in SG from mice with experimental Sjögren’s syndrome (ESS) (66). IL-17 knockout mice immunized with SG proteins did not develop SS (64). The pivotal function of IL-17 in SG inflammation has been extensively studied. However, little is known regarding its effect on SG fibrosis.

Early studies have reported that IL-17 and IL-22 are engaged in the process of EMT (53). Mesenchymal signatures replace the epithelial features during EMT-dependent SG fibrosis in SGECs under the control of IL-17 and IL-22 (53). SGECs treated with IL-17 and IL-22 showed a reduction in cell-cell contacts and adopted a more stretched morphological structure. Furthermore, the levels of IL-17 and IL-22 are associated with focus scores in patients with pSS (67). These findings provide evidence for the importance of IL-17 and IL-22 in the pathogenetic fibrosis of SG. As previously described, IL-17 modulates EMT of epithelial cells depending on TGF-β1 via the SMAD pathway or the noncanonical TGF-β1/Erk1/2/EMT pathway. Among the various subsets of IL-17, IL-17A is known to activate the myofibroblast phenotype that is the initial step in the fibrotic process (68). Proposed treatments for pSS have included those targeting Th17 cells. A proteasome inhibitor bortezomib (BTZ), which targets low-molecular-weight protein 7 (LMP7), demonstrated a notable reduction in IL-17^+^ Th17 cells in ESS mice (69). The monoclonal antibody rituximab has been confirmed to be effective in decreasing the levels of IL-17 (70). Unfortunately, there is a paucity of research focusing on the direct treatment of SG fibrosis via the targeting of Th17 cells and related cytokines.

Similar to IL-17, IL-22 secreted by Th17 cells is also initiated by TGF-β1 with the presence of IL-6 and other cytokines (71). With respect to the function of IL-22 derived from Th17 cells in fibrosis, it was found that this cytokine induces the accumulation of TGF-β1, α-SMA and collagen that favor liver fibrosis (2). IL-22 primarily functions on stromal cells and epithelial cells, playing an important role in tissue regeneration. More importantly, IL-22 also participates in the formation of TLS through the activation of autoreactive B cells (72). IL-22 upregulates the expression of CXCL13 in stromal fibroblasts and CXCL12 in epithelial cells (43). These chemokines promote the B cell aggregation and autoantibody production, which in turn facilitates the TLS assembly. These findings render IL-22 noticeable in SG fibrosis. In addition to IL-17 and IL-22, Th17 cells simultaneously stimulate the release of IL-6, TNF and matrix metalloproteinase 9 (MMP9) in the inflamed SG, exacerbating the local damage and leading to SG fibrosis (73, 74). Moreover, Th17 cells differentiate into a Th17.1 subpopulation that produces IL-17 and IFN-γ or a Th1-like subset, thereby rendering Th17 cells more responsible for the chronic inflammation and fibrosis of SG (75).

#### 2.1.5 Tfh cells

The Tfh subset helps B cell migration to germinal centers and the formation of TLS, which may indirectly support the development of fibrotic structures within the SG (16, 76). In patients with pSS, accumulated data shows that the frequency of Tfh cells in the peripheral blood and in SG is positively linked to disease progression (61, 77). Tfh cells are characterized by the expression of CD40L, CXCR5, PD-1, and inducible co-stimulator (ICOS) (78). An increase in circulating Tfh cells has been observed in patients with IPF associated with SS (16, 79). The pathogenesis of pSS posits that CD4^+^T cells largely differentiate into Tfh cells to augment the number of autoreactive B cells and encourage the generation of ectopic germinal centers (76). The emergence of these germinal centers indicates a significant immune cell infiltration and underlies SG fibrosis. Therefore, further investigation is warranted to elucidate the pro-fibrotic role of Tfh cells in SG. IL-21 is a Tfh-associated cytokine that has been proven to enhance the expression of IL-4Rα and IL-13Rα on macrophages (40). This may serve to reinforce the pro-fibrotic activity of macrophages. In patients with systemic sclerosis (SSc), skin fibrosis could be effectively controlled under the treatment with anti-IL-21 and anti-ICOS antibodies (80, 81).

The blockade of ICOS in SG results in a reduction of both pro-inflammatory IL-6 and TNF-α (82). IL-6 provides essential Tfh-trophic signals and induces IL-21 production (83). In a study by Pontarini et al. (2020), it was demonstrated that IFN-γ is produced by PD1^+^ICOS^+^Tfh-like cells, apart from Th1 cells in patients with pSS (82). The indirect effect of circulating Tfh cells in SG fibrosis can be reflected by the secretion of IL-17, given that IL-17 is highly correlated with SG fibrosis (84, 85). These Tfh-like cells were characterized by CCR9^+^CD4^+^ and CXCR5^+^CD4^+^ phenotype (86). CCR9^+^ Tfh cells specifically express Bcl-6, ICOS and other inflammatory mediators, including IL-7, IL-21, and IFN-γ (86). In ectopic germinal centers, CXCR5^high^CCR7^low^ Tfh cells are likely to migrate to the CXCL13-enriched B-cell follicles, thereby favoring the expansion of autoreactive B cells (86–88). This specific population also shows the ability to regulate the transition from fibroblast to myofibroblast (86). The principal functions of Tfh cells in SG pathological inflammation are manifested in the maintenance of B cell hyperactivity and the induction of memory B cells via IL-21, CD40L, PD-1, Bcl-6 and BAFF (78, 89). This may also suggest their potential involvement in SG fibrosis. T follicular regulatory (Tfr) cell represents a subtype of Treg cells that inhibit the activity of B cells and Tfh cells (78). Recent investigations have also indicated that the ratio of Tfr/Tfh cells may be closely relevant to the severity of pSS through regulation of the formation of ELS (90).

#### 2.1.6 CD8^+^T cells

Although the majority of the infiltrated T cells in SG of SS patients are CD4 positive, the frequency of CD4^+^ T cells is decreased along with the progression of the disease (16). Meanwhile, the number of CD8^+^ T cells was identified to be either stable or even slightly elevated in severe tissue damage (16). This suggests that CD8^+^ T cells may play a role in the inflammatory process of pSS. Indeed, the SG of SS patients displays overactivity of CD8^+^ T cells (91). CD8^+^ T cells are known to exert cytotoxic functions, which eliminate the infection via the Fas/Fas ligand (FasL) pathway, leading to the large-scale cell apoptosis and tissue damage (**Figure 1**), including acini injury (91). In the vicinity of apoptotic epithelial acinar cells, CD8^+^ T cells accumulate in an aberrant manner and release cytotoxic-related molecules, including IFN-γ, TNF-α, perforin, and granzyme B (92). The recruitment of activated CD8^+^ T cells may owe to CXCR3-mediated chemotaxis, which drives the CD8^+^T cells’ migration into the inflamed SG in patients with pSS (92).

CD8^+^ T cells with tissue-resident memory (TRM) signature have been recently highlighted in the contribution to tissue fibrosis in aged mice (93). Single-cell transcriptomics of SG-infiltrated and peripheral blood immune cells have depicted that CD8^+^CD9^+^ T cells constitute the tissue-resident memory population infiltrated in SG biopsies of pSS patients (94). In both ESS mouse models and patients with pSS, tissue-specific CD8^+^CD103^+^CD69^+^ TRM was identified as being involved in sialadenitis, with a significant increase in IFN-γ production, in which CD69 and CD103 are the tissue residence markers (95). This TRM population was found to be remarkably expanded and activated in the local SG of ESS mice and patients with pSS (95). When performing anti-CD103 treatment in experimental mice, the collected data showed the restoration of SG functions and a reduction in immune cell aggregations, as evidenced by the focus score (P<0.01) (95). Concomitantly, the release of CD8-related cytokines was also significantly reduced. Interestingly, the results of immunohistochemistry staining presented a negative correlation between TRM proliferation and the expression of epithelial phenotypic markers, such as E-cadherin. This finding may indicate a close association between CD8^+^-specific TRM and SG fibrosis.

Recent studies by Gao et al. used P40^-/-^CD25^-/-^mice as a murine model of SS to investigate the contribution of CD8^+^ T cells (96, 97). P40 chain is an essential component of IL-12 and IL-23 (98). Both of them are essential for maintaining the equilibrium between Th1/Th17 (98–100). IL-12 is engaged in the Th1 response, while IL-23 is responsible for the Th17 response. P40^-/-^CD25^-/-^mice were identified as being able to develop SS and exhibiting distinct lymphocytic foci, atrophy and fibrosis in SG (96). In this mouse model, CD8^+^ T cells are the predominant infiltrating lymphocytes in the inflammatory site, and are accompanied by TRM features and high IFN-γ production (96). CD8 knockout mice exhibited a significant reduction in the severity of the inflammatory lesion in SG including fibrosis (96). Consequently, it can be postulated that this CD8^+^ TRM subpopulation may be responsible for fibrotic morphological change in P40^-/-^CD25^-/-^mice. However, this finding may be limited to the specific mouse model, given the fundamental functions of P40 and CD25 in immune tolerance. The absence of CD4^+^ T lymphocytes in mice is an atypical phenomenon and cannot mimic the real situation in humans.

A recent breakthrough in research has revealed that the single-cell gene expression profile of granzyme K^+^ (GZMK^+^) CXCR6^+^CD8^+^ T cells in peripheral blood is comparable to that of CD69^+^CD103^–^CD8^+^ TRM in SG of pSS (101). The circulating GZMK^+^ CXCR6^+^CD8^+^ T cells, particularly those with high expression of CD122, may be the reservoir of CD8^+^ TRM subpopulation in tissue (101). IL-15 is a key factor in the differentiation of CD8^+^ T cells into GZMK^+^ CXCR6^+^CD8^+^ T cells. Importantly, with respect to CD8^+^CD69^+^ TRM, the CD103^-^ subset appears to be more pertinent to chronic inflammation of SG due to its elevated level of GZMK, in comparison to the CD103^+^ subset (101). Moreover, the CD103^-^ subset exhibits heightened sensitivity to T cell receptor signaling and is capable of producing greater quantities of pro-inflammatory cytokines such as IFN-γ and TNF-α, which are associated with the severity of inflammation in SG as well as fibrosis.

### 2.2 B cells

Numerous studies identified autoreactive B cells as important participants in the pathogenetic process in SS. Based on the current knowledge, they are predominantly involved in the later stage of the disease. Abnormal B cell response to autoantigens Ro/SSA and La/SSB renders B cells differentiation into plasma cells and produces excessive autoantibodies (102). The accumulation of these autoimmune-related antibodies further advances chronic inflammation in the epithelium of the exocrine glands, which may outcome glandular fibrosis (103, 104). The formation of lymphoepithelial lesions (LELs) is initiated by T cell infiltration in the ductal epithelium (105). The majority of B cells in duct epithelium of SG appear after T cells and are derived from the recruitment of T helper cells. Attracted B cells are present in approximately 60% of SG in pSS patients (105). However, in contrast to T cells that exist in almost all ducts of SG in both pSS and non-SS patients, the B cells that infiltrate the ducts with LELs are more specific to this particular type of lesion (105). The diverse functions of B cell subsets have been acknowledged in the context of autoimmune pathogenesis (106). Epithelium-associated Fc Receptor-Like 4 (FcRL4)^+^ B cells have been identified as a pathogenic cell type that contributes to the development of pSS, with an increased secretion of pro-inflammatory factors that support the formation of LELs, such as IL-6 (107). Age-associated CD11c^+^ B cells have also been observed to produce SS-related cytokines, including IL-1β, IFN-γ and IL-10 (106). Marginal zone B cells (MZ B) have been found to accumulate in SGs and are linked to the production of autoantibodies that cause epithelial destruction (108, 109). The autoreactive BCR may be associated with the MZ phenotype (110). In addition, multiple regulatory mechanisms have been proposed to explain the migration and infiltration of B cells into inflamed glands. CXCR3 and CXCL10 attract B cells into an ectopic germinal center (28, 29) (**Figure 1**). Ectopic germinal center is the local center for the activation and maturation of autoreactive B cells. A substantial number of immune cells within ectopic germinal center contributes to SG chronic inflammation and is considered a defining feature of SG fibrosis (81). In patients with SS, serum levels of IL-22 are closely associated with saliva flow and autoantibody production (44). IL-22 can activate the CXCL13 in stromal fibroblasts, and subsequently facilitate the ELS assembly via the CXCR5/CXCL13 axis (43). As Tfh cells mainly express the CXCR5, IL-22 indirectly influences the regulation of T cell-dependent B cell hyperactivity by inducing CXCR5^+^ Tfh cell migration to B cell follicles (43, 44). Tfh cells critically support B cell survival, maturation, and the aggregation of memory B cells, mediated by CD40L, IL-21, and BAFF (102, 111). Similarly, IL-17 has been shown to orchestrate the establishment of the autoreactive germinal center in autoimmune mouse models (112). IL-17, when present alone or in conjunction with BAFF, can support the expansion of autoreactive B cells (113). Collectively, several factors involved in the interaction of B cells and ectopic germinal centers simultaneously promote the development of chronic inflammation-dependent fibrosis in SG.

Notably, regulatory B cells (Breg) are defined as immune suppressors of autoimmune diseases in an IL-10-dependent manner (114). The role of Breg cells in the development of sialoadenitis fibrosis remains largely unclear. Breg cells are capable of inhibiting the trophic signals of Th1 and Th17 responses which are indispensable in the pathogenesis of autoimmunity (115). Bregs also stimulate the expansion of Treg cells and suppress inflammation (114). More importantly, IL-10-producing Breg cells have been identified as markedly repressing the Tfh response by stimulating the STAT5 phosphorylation, either in patients with pSS or ESS in mice (115). Together, Breg cells are able to effectively regulate the development of inflammation and germinal center formation by inhibiting Th1, Th17 and Tfh responses, thus exerting an anti-fibrotic effect. However, they are also capable of promoting Treg proliferation, which may lead to upregulation of the expression of the pro-fibrotic factor TGF-β. More research is needed to evaluate the function of Breg cells in SG fibrosis.

### 2.3 Macrophages

Macrophages are a critical cell type in the programming of tissue repair and fibrosis. They play a dual role in the initiation of the repair process and the maintenance of ECM within the tissue (116). In response to tissue injury, macrophages differentiate into versatile phenotypes, including tissue resident macrophages, which are responsible for wound healing (39). Systemic repair signals instruct macrophages to rebuild tissue with normal architectural structure (37). Nevertheless, prolonged tissue damage can result in abnormalities of macrophages, which may lead to irreversible morphological changes and pathological fibrosis. Macrophages have the plasticity to transition between M1 and M2 phenotypes, contingent upon the prevailing immune milieu. For instance, IL-4Rα signaling stimulates the M2 phenotype that determines liver inflammation and fibrosis in SSc (81, 117). Tissue injury frequently drives the polarization of macrophages into the pro-inflammatory M2 phenotype, which is characterized by the release of large amounts of cytokines, such as IFN-γ, TNF-α, IL-4, IL-13, IL-10 and TGF-β (36, 116, 118–120). These cytokines have been confirmed to contribute to the SG fibrosis by enhancing the local chronic inflammatory environment and inducing the activation of myofibroblasts, especially the macrophage-derived TGF-β (121).

TGF-β is involved in the EMT-dependent fibrotic signaling pathway in SG, namely the TGF-β1/SMAD/Snail pathway as previously described. This pathway is a key factor in the progression of fibrogenesis. Hypoxia-inducible factor-1α (HIF-1α) has been identified as being engaged in the fibrogenesis triggered by TGF-β1 (122). HIF-1α is a transcription factor that regulates oxygen homeostasis. This finding generates a link between fibrosis and tissue hypoxia (**Figure 1**). The knockout of the HIF-1α gene resulted in a reduction in TGF-β1 expression in alveolar macrophages, thereby alleviating bleomycin-induced fibrosis (37). The activation of latent TGF-β stored in the ECM by CD14^+^ monocyte-derived macrophages via integrin αvβ8-mediated pathways has been demonstrated (123). In addition, macrophages are able to influence heterogeneity of myofibroblasts through interactions with the ECM (39). Macrophages are also the primary source of MMPs, a family of enzymes responsible for ECM degradation in tissue (124). MMPs are important contributors in fibrosis and their functions will be discussed in the following section. To this end, elucidating the mechanism by which macrophages induce pathological fibrosis is advantageous for future therapies to regulate the progression of SG inflammation and fibrosis in patients with SS.

### 2.4 Mast cells

Mast cells are a type of resident immune cell that are mainly found in connective tissues. They primarily play a role in allergic reactions by releasing inflammatory mediators such as histamine and heparin (125, 126). Numerous studies have indicated their functions on tissue repair and chronic inflammation in autoimmune diseases. Their pro-fibrotic function in SG has recently been revealed (12). The interplay between mast cells and fibroblasts stimulates MMPs production, which have been shown to be positively associated with tissue damage (127). The infiltration of mast cells is elevated in the fibrotic zone of SG tissue from pSS patients (125). In this study, the researchers observed a significant correlation between the number of mast cells and the levels of fatty infiltration and fibrosis in minor SG of 19 patients with pSS and 10 healthy individuals (125). Mast cells respond to the acute phase of pSS and release mediators such as IL-1β, TNFα and IL-33 (126), which enhance the focal inflammation in SG. In addition to its capacity to stimulate the Th2 response, IL-33 exhibits pro-inflammatory properties that favor pathological development (118, 128). Moreover, mast cells can interact with fibroblasts through IL-33 (129). This interaction may exacerbate the autoreactive T cell response in the local tissue. The potential role of mast cells in the fibrogenic program could be exploited therapeutically.

### 2.5 Neutrophils

Multiple recent studies implicated that neutrophils are essential in the pathogenesis of autoimmune disorders, including RA, SLE, and pSS (130). The release of inflammatory cytokines by neutrophils has been identified as a key mechanism by which these cells contribute to the progression of autoimmune diseases (130). An increased basal adherence and increased random migration of neutrophils have been observed in patients with pSS (131), indicating the activation of blood neutrophils and a correlation with an elevated risk for recurrent bacterial infections (132). Upon encountering pathogens, neutrophils undergo degranulation and produce reactive oxygen species (ROS), subsequently entering the NETosis process, which is a form of programmed cell death (133). It is also important that neutrophils have the capacity to migrate from the circulation into resident tissue, where they produce MMPs as one of the antimicrobial peptides that prevent pathogen invasion (134). This may indicate their involvement in ECM degradation. In patients with pSS, neutrophils produce ROS-related molecules during the respiratory burst (135), which may be relevant to the occurrence of inflammation and fibrosis. The results of RNA sequencing and qPCR from a recent study have been corroborated by the observation that neutrophils in patients with pSS exhibited aberrantly elevated expression of genes associated with the type I interferon pathway (136). Moreover, the hyperactivity of type I IFN genes results in mitochondrial damage and is accompanied by ROS activation which favors the formation of neutrophil extracellular traps (NETs) (136, 137). NETs are the components released from NETosis. The generation of NETs may increase the production of autoantibodies (137–139) and cause tissue damage in the context of autoimmune response (137, 138). In conclusion, the impact of neutrophils on SG fibrosis may be attributed to the activation of the type I IFN pathway and MMP-related or ROS-related tissue damage.

## 3 The pro-fibrotic role of non-immune cells in salivary gland of pSS

### 3.1 Fibroblasts

**Figure 2:**
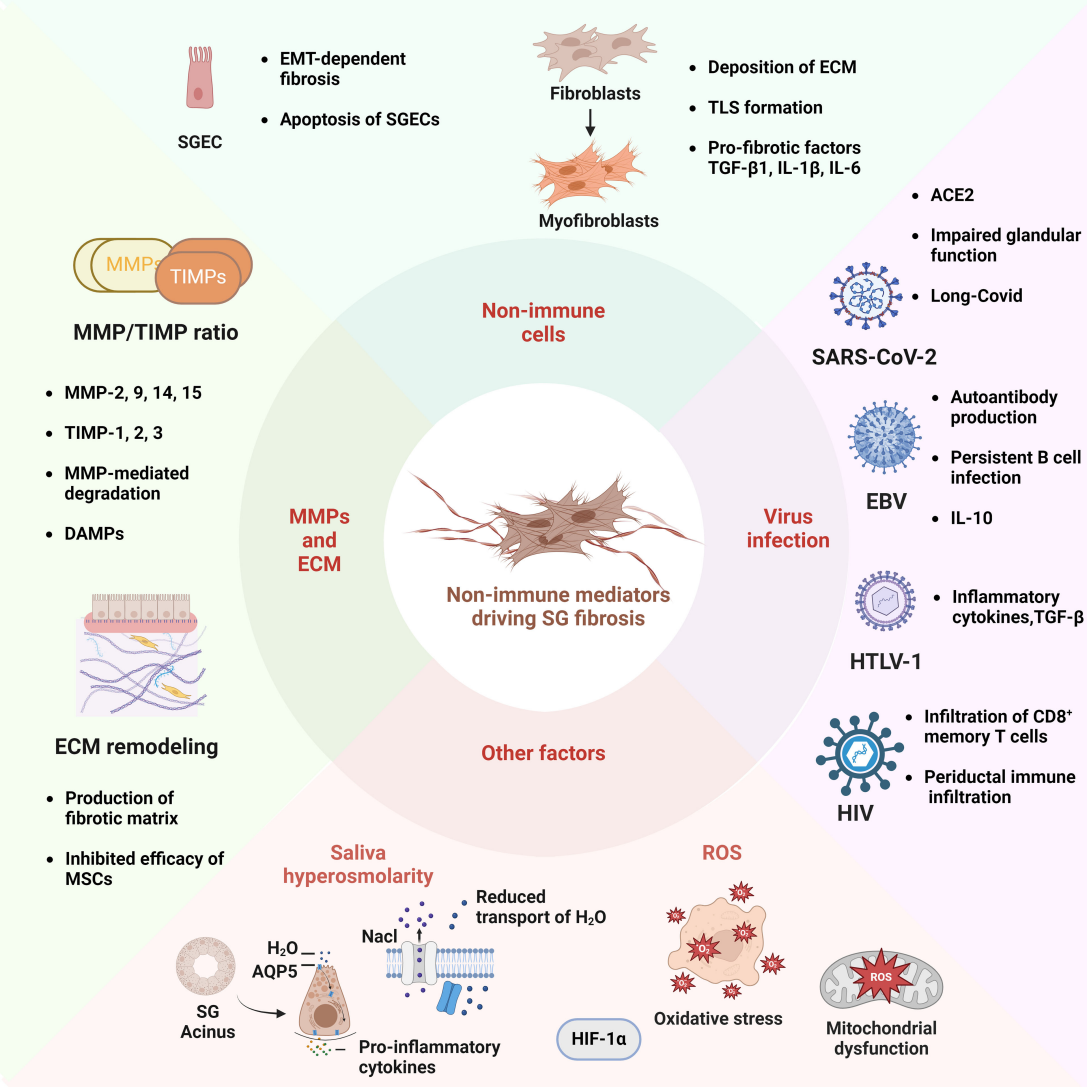
Schematic Illustration of Non-Immune Mediators Associated to the Fibrotic Pathogenesis of Sjögren’s Syndrome. In terms of non-immune factors, the EMT and apoptosis of SGECs are critical processes in the development of salivary gland fibrosis. The transformation of fibroblasts into myofibroblasts is an important step in the development of SG fibrosis. This process involves the deposition of ECM, the formation of TLS, and the accumulation of inflammatory cytokines. The equilibrium between matrix MMPs, such as MMP-2 and -9, and tissue inhibitors of metalloproteinases (TIMPs), including TIMP-1 and -2, influences the degradation of ECM and facilitates the accumulation of fibronectin. The mis-localization of the aquaporin 5 (AQP5) water channel protein in SGECs may result in increased saliva osmolarity, which in turn impairs water transport, while simultaneously enhancing the production of pro-inflammatory cytokines. Viral infection represents a significant etiological factor in SS, with the SARS-CoV-2 virus, Epstein-Barr virus (EBV), human T-lymphotropic virus (HTLV-1), and human immunodeficiency virus (HIV) serving as prominent examples. Additionally, the process of SG fibrosis is influenced by other mechanisms, including oxidative stress and mitochondrial dysfunction. The diagram was created using BioRender.com.

Fibroblasts manage the tissue regeneration and homeostasis through the production of connective tissue components, including fibronectin and collagens (140). The differentiation of fibroblasts into myofibroblasts is widely recognized as an essential step in driving tissue fibrogenesis in autoimmunity (141). In particular, fibroblasts in SG have been reported to elicit immunomodulatory functions in pSS (**Figure 2**). SG-resident fibroblasts participate in the organization of TLS, which is relevant to the level of inflammation and the severity of pSS (42). The TLS structure is well-organized, exhibiting distinct immune cell aggregations and high endothelial venules (72). The fibroblastic network in SG has been proven to be initiated and expanded by the IL-13 and IL-22. Myofibroblasts are typically contractile and α-SMA positive, enabling the release of pro-fibrotic factors such as TGF-β1, IL-1β, IL-6, vascular endothelial growth factor (VEGF), and IL-33 (38). Myofibroblasts regulate not only the deposition of ECM but also the EMT process, thereby driving tissue fibrosis.

In a single-cell gene expression profile of the SG in pSS patients, CXCL10^+^ CCL19^+^ fibroblasts interact specifically with immune cells to establish TLS (142). CXCL10^+^ CCL19^+^ immuno-fibroblasts have a similar effect as podoplanin (pdpn)^+^ CD34^−^ CCL19 expressing fibroblasts in the context of TLS formation (142). The interplay between T cells and dendritic cells is promoted by pdpn-expressing fibroblastic reticular cells in conjunction with pdpn^+^ stromal fibroblasts, facilitating the transition from innate to adaptive immunity within TLS (38, 142, 143). In patients with SS, CXCL10^+^ CCL19^+^ fibroblasts may participate in the development of inflammatory lesions by providing signals such as IFN, IL-1, and TNF, which could exacerbate SG lymphocytic infiltration and favor the resemblance of TLS (31, 38, 142). Single-cell sequencing data has also revealed the existence of another distinct pathology-associated fibroblast subtype in chronic inflammatory diseases, namely secreted protein acidic and cysteine-rich (SPARC)^+^ COL3A1^+^ fibroblast (142). SPARC^+^ COL3A1^+^ fibroblasts can interact with vascular endothelium and thereby support the ECM restoration. The attenuation of fibrosis via SPARC siRNA has been documented (144). Thus, therapeutic strategies aiming at targeting pathogenic, inflammation-associated fibroblasts are considerable to alleviate the SG fibrosis process.

### 3.2 SGECs

In patients with pSS, the loss of epithelial character and acquisition of a mesenchymal signature have been well documented during chronic inflammation and fibrogenesis of SG (51). TGF-β is able to induce epithelial cell plasticity in response to inflammatory signals (50). Given the importance of TGF-β in the advancement of EMT-dependent fibrosis in pSS, as a vital fibrogenic mediator, it activates and phosphorylates the canonical TGF-β1/Smad2/3 or non-canonical TGF-β1/Erk1/2 pathway in SGECs, thus mediating their transition to mesenchymal phenotype with a myofibroblast-like morphology. It is noteworthy that IL-6 has been depicted to markedly induce EMT-dependent SG fibrosis in pSS (56). When human SGECs are cultured with IL-6 for 24 to 72 hours, a downregulation of epithelial markers, an upregulation of mesenchymal markers, and a clear morphological change to a mesenchymal phenotype can be observed (56). Additionally, it has been identified that IL-7, secreted by local SGECs, enhances Th1 responses (145). IL-7 also modulates the level of TNF-α, a prototypical Th1 cytokine that contributes to the perpetuation of chronic inflammation in SG (146–148).

As atypical antigen-presenting cells, SGECs play a crucial role in mediating local autoimmune responses (149). SGECs interact with B cells to coordinate differentiation into plasma cells and promote the production of autoantibodies (150). Abnormal interactions between SGECs and immune cells contribute to the exacerbation of local tissue injury in SG. The presence of TNF and IFN-γ in pSS induces apoptosis of SGECs via the Fas/FasL pathway (151). IFN-γ-induced or poly (I:C)-induced anoikis results in the impairment of secretory gland function and high titers of autoimmune antibodies in serum (152). Apoptotic cells also release exosomes that cause sustained damage to the epithelium (153). In conclusion, SGECs are deeply implicated in the pathogenesis of chronic inflammation in SG and may also influence SG fibrosis (**Figure 2**).

## 4 Other pro-fibrotic mechanisms in salivary gland of pSS

It is evident that additional molecular mechanisms may contribute to the development of fibrosis in SG of pSS. In the following sections, three distinct factors that contribute to SG fibrosis will be mainly discussed in detail: MMPs and ECM remodeling, salivary hyperosmolarity, and viral infection. It should be noted, however, that there are other molecules and pathways that are relevant to SG fibrosis, although they have been previously only briefly mentioned in the immune cell section.

### 4.1 MMPs and ECM remodeling

A glandular response to tissue disruption typically gives rise to an altered extracellular matrix. In the context of pSS progression, SG destruction is common due to the chronic inflammatory environment and ultimately leads to the formation of fibrotic matrix. MMPs are a family of enzymes that are capable of degrading matrix. MMP-2, 9, 14, 15 and tissue inhibitors of metalloproteinases (TIMPs)-1, 2, 3 are all involved in the homeostasis of SG ECM (127, 154, 155). The dysregulation of MMPs drives ECM remodeling and SG degeneration following sequential pathological events, inducing the production of fibrotic matrix (156). The fibrotic basal lamina displays a disorganized pattern of laminin expression (157). A fibrotic matrix impedes the transportation of nutrients and oxygen to the cells and blocks the infiltration of progenitor cells, which are required for tissue renewal (158). Indeed, fibrotic ECM impacts the efficacy of SG regenerative therapies, especially the therapeutic effects of mesenchymal stem cells (MSCs) (158, 159). The activity of MMPs can be regulated by TIMPs (127) (**Figure 2**). Among these MMPs and TIMPs, the upregulation of MMP-3 and MMP-9 and downregulation of TIMP1 are positively correlated with acinar damage and disease development in pSS patients (127, 160). The ratio of MMPs/TIMPs in human SG is one of the hallmarks of ECM remodeling. A number of pro-inflammatory cytokines are responsible for mediating the balance between MMPs and TIMPs, including IL-1α, IL-6, TNF-α, and IFN-γ (124, 160, 161). The process of anoikis of cells also enables the activation of these enzymes. Additionally, MMP-mediated degradation in SG produces the damage-associated molecular patterns (DAMPs), which subsequently activate the Toll-like receptor 2 (TLR2) and TLR4 pathways (162). Consequently, the nuclear factor kappa B (NF-κB) pathway is activated, further sustaining the chronic B cell response *in situ*. For example, the small proteoglycans, biglycan (Bgn) and decorin (Dcn) are present in the damaged SG basal lamina in SS mice (162). As components of the ECM and typical DAMPs, these two proteoglycans can bind to TLR4, which subsequently elicits MyD88-dependent signaling cascades, thus exacerbating the downstream autoimmune responses (162). Increased anti-Bgn and -Dcn autoantibodies in serum were found in experimental mice of SS and were correlated with SG dysfunction (163). Besides, aging may also be risk factor in ECM remodeling in SG. Recent studies have revealed that accumulated MMP-2 and MMP-9 are strongly connected with age-related fibrotic remodeling, particularly in aged mice exhibiting a senescence-associated secretory phenotype (SASP) (158, 164).

### 4.2 Saliva hyperosmolarity

Dysfunctional exocrine glands are unable to produce saliva of the requisite quality and quantity. The displacement of the aquaporin-5 (AQP-5) water channel protein suggests that water is not properly and adequately transported out of the apical membrane (165, 166) (**Figure 2**). In contrast, the transport of NaCl to the acini lumen results in the formation of saliva with a high osmotic gradient (167). Since acinar cell apoptosis could cause a reduction of salivary volume, the formation of a hyperosmolar environment within the salivary gland could potentially lead to further remodeling of ECM and severe lymphocytic infiltration of SG (167). Notably, gene enrichment analysis of acinar SG cells in response to hyperosmolar stimulation showed robust pro-fibrotic gene expression such as *IFN-γ*, *IL-17, MMP-3*, and *MMP-13* (167). SGECs from pSS patients showed overexpression of MMP genes that were related to ECM regeneration including *COL3A1* and *MMP-24*, compared to healthy controls (142, 167). Of note, this was associated with upregulation of Snail in SGECs by RNA-seq analysis, which indicates the EMT process in SG. These findings suggest a possible implication of hyperosmolar stress in the pathogenesis of fibrosis.

### 4.3 Viral infection

It is well known that the tissue fibrosis can be the result of prolonged inflammation in response to a multitude of stimuli, including viral infections, radiation and immune hyperactivity. In studies about the etiology of pSS, several virus infections have been proposed as potential drivers of chronic persistent inflammation and autoimmune response in SG (168, 169). These include the Epstein-Barr virus (EBV), hepatitis C virus (HCV) and human T-lymphotropic virus (HTLV-1) (**Figure 2**) (169–171). The ability of viral antigens to drive autoimmunity may be, at least in part, due to their molecular mimicry of autoantigens (172–174). These viral antigens possess analogous sequences and structures, favoring cross-reactivity of immune cells with autoantigens and the initiation or promotion of overproduction of autoantibodies (174). *In vivo* studies have reported the role of viral infection in the mouse salivary gland model. Replication-deficient adenovirus-5 (Adv5) was introduced into the submandibular glands of C57BL/6 mice by retrograde cannulation (175). Adv5 infection may result in reduced saliva, exocrine dysfunction, autoantibody production and TLS formation. The upregulation of CXCL13, CCL19 and CCL21 mRNA accelerated the development of ELS (176). This finding suggests that viral infection indeed impairs SG function and contributes to the organ-specific autoimmune pathogenesis.

The precise role of various viral infections in the pathogenesis of fibrosis in the salivary glands of patients with SS remains incompletely understood. EBV is particularly associated with the onset and progression of SS, as it has been demonstrated to trigger or promote autoimmune responses and autoantibody production (174, 177). EBV is capable of infecting both naive and memory B cells, and it can persistently infect those B cells (178). EBV has been demonstrated to promote B cell differentiation into plasma cells and to increase the production of autoantibodies (179). Furthermore, sustained EBV infection can damage the SG due to IL-10 production, which also results in the production of autoantibodies (180). Taken together, it remains unclear whether EBV plays a pro-fibrotic role, but it is evident that it can accelerate the progression of SS. Additionally, other viruses, including HCV, Hepatitis B Virus (HBV), and HTLV-1, have been identified as potential contributors to the pathogenesis of SS. HCV infection is primarily associated with hepatic impairment in SS (181, 182). While hepatitis and liver fibrosis are well-documented consequences of HCV infection, the pro-fibrotic effect of HCV on the SG is less frequently observed. HBV is more closely associated with liver involvement. HTLV-1 infection has been identified to enhance the production of inflammatory cytokines, including TGF-β, which is associated with fibrosis in SG tissues of SS patients (174, 183). Interestingly, a reduction in ectopic germinal centers has been observed in HTLV-1-seropositive SS patients, which may be attributed to its inhibitory effect on B cell-related cytokines, such as BAFF (184). Human immunodeficiency virus (HIV)-related SS is characterized by the infiltration of CD8^+^ memory T cells and the presence of viral proteins, including LMP-EBV and p-24, in ductal cells (185). A recent histochemical study demonstrated that acinar atrophy, ductal dilation, and fibrosis, accompanied by collagen deposition in the site of periductal immune infiltration, are hallmarks of HIV-related SS (186, 187). In general, viral infections may contribute to the development of fibrosis in SG through various mechanisms, including the induction of cytokines, chemokines, and interaction with immune cells.

Interestingly, SARS-CoV-2 may also contribute to the pathological process of SS (**Figure 2**). Angiotensin-converting enzyme 2 (ACE2) transgenic mice were infected with SARS-CoV-2. The researchers found distinct SS-like signatures such as impaired glandular function, elevated anti-SSB/La and severe lymphocytic infiltrates in the SG of infected mice and individuals diagnosed with SARS-CoV-2 infection (188, 189). Clinical data have shown that the 2019 novel coronavirus (2019-nCoV) is detectable in the saliva of 91.7% of infected patients (190). ACE2 is the target receptor of SARS-CoV-2 and is present in the SG. As evidenced by research, ACE2 is mainly expressed in epithelial cells within SG (191). Thus, it can be concluded that SGECs are the target cells during SARS-CoV-2 infection. More importantly, SARS-CoV-2-mediated tissue pathology is frequently accompanied by lymphocytic lesions due to the local auto-reaction (192, 193). These findings imply a potential role for SARS-CoV-2 infection in the development of pathogenic fibrosis within the salivary gland. While there have been reports of lung fibrosis associated with SARS-CoV-2 infection, there is a paucity of research on the topic of salivary gland fibrosis. Therefore, further investigation with a larger cohort of patients with chronic inflammation is necessary to gain a deeper understanding of the relationship between SARS-CoV-2 infection and the development of fibrosis.

### 4.4 Other potential factors

In addition to the above mechanisms, there are other non-immune factors that contribute to SG fibrosis, such as oxidative stress and mitochondrial dysfunction. Cyclophosphamide (CP), a chemotherapeutic agent which is also used as an immunosuppressant causes oxidative stress (194, 195). However, it can affect SG and reduce saliva production in a long-term manner. CP can increase the expression of oxidative genes and at the same time induce the morphological changes such as parenchymal atrophy and acinar apoptosis of SG (195). In addition, inflammatory markers such as TNF-α and IL-1β were increased in the periductal area induced by CP application. 5-Fluorouracil (5-FU) is also a drug used to treat solid tumors (196). Similar to CP, it can activate the oxidative system and lead to the lesions and morphological alterations of SG such as atrophy and necrosis (195, 196). The treatments of CP and 5-FU may induce inflammation in SG via oxidative stress, and ultimately aggregate the progression of SG fibrosis. ROS are mainly generated by mitochondria via oxidative phosphorylation. Damaged mitochondria produce more ROS and increase the levels of oxidative stress markers such as 8-OHdG and HEL in patients with pSS (197). Furthermore, the impairment of the cell leads to DAMPs molecules released by mitochondria, following the immune reaction cascades (198). Impaired mitochondrial respiratory chain produces less ATP, which exacerbates cell apoptosis and recruits immune cells (198). Thus, mitochondrial dysfunction may play a role in the immune cell infiltration and inflamed microenvironment of pSS, increasing the oxidative stress and resulting in SG fibrosis.

## 5 Therapeutic strategies

A variety of treatments are available for the alleviation of sicca symptoms, including the use of saliva substitutes. Systemic secretagogues pilocarpine and cevimeline, which act as muscarinic receptor agonists, are capable of relieving symptoms of dry mouth (1). Several monoclonal antibodies specifically targeting B cells have shown favorable outcomes, including rituximab (a CD20­specific antibody), epratuzumab (a B cell receptor CD22-specific antibody) and belimumab (a BAFF-blocking antibody) (199–201). With regard to the treatment for SG fibrosis, research is now concentrating on tissue regeneration and anti-inflammation. An siRNA was effective in knocking down the transcription factor of MMP-9, namely ETS1, showing beneficial effects in MMP-mediated degradation (202). A chimeric antigen receptor (CAR)-T cell targeting fibroblast-activating protein (FAP) has been proposed as a potential treatment for oral submucous fibrosis (OSMF) (203). FAP^+^ fibroblasts possess a greater potential for conversion into myofibroblasts. Therefore, this CAR-T cell may be an effective means of potentiating an anti-fibrotic approach. In addition, microRNAs targeting the various components of the TGF-β1/Smad pathway have been shown to be effective in inhibiting TGF-β1-dependent tissue fibrosis (204).

To date, regenerative therapies remain as the mainstay in the treatment of tissue fibrosis. MSCs treatment has been demonstrated in numerous studies to effectively suppress pathogenic processes in SS and influence the SG microenvironment by replacing the fibrotic matrix (158, 160, 205–208). A number of different studies have demonstrated the efficacy of MSC administration in the treatment of SG (205–208). Injection of MSCs resulted in a significant increase in salivary flow rate and decreased lymphocytic infiltration in the SG in a mouse model of the disease (205, 209–211). According to the study by Xu et al., the levels of anti-SSA/Ro and anti-SSB/La antibodies were downregulated after injection of umbilical cord MSCs in 24 patients with SS (206). In patients with SS, administration of MSCs effectively reduces the level of IL-12, a pro-inflammatory cytokine produced by DCs and implicated in the development of SS (212). Treatment with MSCs may affect the differentiation and maturation of DCs, resulting in the suppression of IL-12 (213). Furthermore, intravenous injection of MSCs in 38 patients showed increased IL-27 levels and decreased ratio of Th17/Treg cells (211). The elevated Treg cells among PBMCs indicated alleviation of SS symptoms (211). These studies provided the clinical evidence for MSC treatment in patients with SS. Moreover, the homing ability to site of tissue regeneration and inflammation makes MSCs remarkable in the treatment of SG impairment. MSCs, as the reservoir of progenitor cells, have the capacity to regulate the SG regeneration. Immunoregulatory cytokines secreted by MSCs reduce B-cell activation and proliferation by inhibiting the overactivation of T cells and dendritic cells (205, 214). MSC can also interact with fibroblasts, thereby exerting an anti-inflammatory effect (215). These cells have the potential to reduce the expression of inflammatory molecules such as intercellular adhesion molecule 1 (ICAM1) and vascular cell adhesion protein 1 (VCAM1) as well as ECM-degrading MMPs (160, 216). Together, these findings suggest anti-fibrotic properties of MSCs by suppressing inflammation and ECM degradation. To investigate the anti-fibrotic activity of MSCs, a previous study conducted by van der Helm et al. (2018) injected bone marrow-derived MSCs (BMMSCs) into a zebrafish embryo model (208). The results demonstrated that liver fibrosis in zebrafish was attenuated in parallel with decreased collagen and TGF-β levels following administration of BMMSCs. Another study reported reduced IL-1β expression and inhibited pro-fibrotic signaling in a cardiac ischemia mouse model treated with MSC exosomes (217). In both mouse models and pSS patients, BMMSCs can promote the immunosuppressive activities of Treg, thereby inhibiting the autoimmune progression and improving the glandular function (208). In an SS-like NOD/Ltj mouse model, treatment with allogeneic bone marrow-derived MSCs showed a therapeutic effect in alleviating SS symptoms and improving the performance of SG (206). The immunoregulatory activities of MSCs are evident in directing the fate of CD4^+^ T cells towards Treg and Th2, while simultaneously suppressing the Th17 and Tfh pathways (206). In particular, researchers have revealed that the migration of BMMSCs to the site of inflammation is controlled by the stromal cell-derived factor-1 (SDF-1)/CXCR4 axis, which determines the functions of MSCs against SS autoimmunity (119, 206, 208). Collectively, these findings suggest the anti-fibrotic capacity of MSCs in diminishing ECM degradation and SG fibrosis. However, MSC therapy is not without limitations and potential adverse effects. Resident MSCs have the potential to differentiate into myofibroblasts. The accumulation of myofibroblasts derived from MSCs may lead to massive ECM deposition and organ failure (218). TGF-β1 secretion by external MSCs can promote myofibroblast populations and immune system abnormalities (219). Furthermore, Ben-David et al. revealed the possibility of tumor formation after MSC transplantation (220). The researchers conducted an analysis of the MSC-specific chromosomal aberrations that may be responsible for the increase in tumorigenicity. In the study by Liang et al., they evaluated the adverse effects of allogeneic MSCs in 404 patients with autoimmune diseases including SLE and SS. They found that the rate of infections caused by MSCs transplantation was 29.5% and the risk of mortality was 11.1% after 9 years of follow-up (221). It is therefore imperative that the safety and fine-tuning of MSCs therapy in SG fibrosis urgently requires further exploration.

## 6 Discussion

This review examines the contributions of immune and non-immune cells to the pathophysiological processes of fibrosis in the SG of patients with SS. Although a large number of studies have proposed risk factors for this autoimmune disease, the mechanism of fibrogenesis and the interplays between SGECs and immune cells in SS remain poorly understood. This morphological alteration is typically irreversible and associated with organ dysfunction. In the early stage of SG fibrosis, reduced acini, architectural malformation and ductal dilation could be observed under the microscope. More tissue injury may induce more FLS formation. The large number of FLS is the standard to evaluate the severity of this disease. These may be the morphologic indicators of SG fibrosis. The molecular mechanism causing these pathomorphologic changes is indispensable with abnormal immune response in the context of SS. An excessive autoreaction in the SG of patients with SS or SjD leads to chronic inflammation in the local area. The persistent inflammation observed in the SG is attributed to detrimental immune infiltration and the accumulation of inflammatory cytokines. TGF-β-mediated EMT may be one of the significant mechanisms contributing to the development of fibrosis in SG. Aberrant T-cell and B-cell responses lead to the overproduction of autoantibodies and aggravate tissue damage. It is therefore evident that immune cells underscore their role in exacerbating chronic inflammation of the SG to the point of progressive fibrosis. As for the non-immune cells, they interact with varying extents with fibroblasts and regulate their transformation into myofibroblasts, the culprits of tissue fibrosis. Among the non-immune fibrogenic mediators, MMPs play a critical role in ECM remodeling. MMPs facilitate the replacement of healthy components of the ECM with fibrosis-associated tissue in response to damage signals. Therefore, the development of SG fibrosis represents a deleterious consequence of an excessive reparative response to damage in the collaboration of immune cells and tissue-resident cells. This response is characterized by the production of pro-inflammatory cytokines and enzymes that degrade the ECM and cause local chronic inflammation. Surprisingly, viral infection, including that caused by EBV, HCV, HTLV-1 and SARS-CoV-2 virus, may also trigger the pathogenic processes in patients with SS. Long-term consequences following recovery from SARS-CoV-2 infection include oral complications such as glandular atrophy and fibrosis. The effect of SARS-CoV-2 on SG gland was unexpected and requires further investigation. As of yet, the influence of oral manifestations and the mechanism of dysfunctional SG in the context of COVID-19 remains unclear.

In fact, SG fibrosis is implied in long-COVID or post-COVID conditions, as fibrous connective tissues are formed to repair the glandular damage (189). Excessive formation of hyperplastic fibrous scar tissue results in the obstruction of the gland. A seminal study by Alfaifi et al. elucidated the long-term oral sequelae in post-COVID patients including salivary reduction, glandular destruction and taste dysfunction (222). Both minor and major SGs displayed SS-like features. These included architectural malformation, atrophy, fibrosis and ductal disruption following SARS-CoV-2 infection (188). An antimicrobial peptide called histatin-5 (Hst-5) is involved in the innate immune response during infection. Decreased salivary histatin-5 production has been implicated in SARS-CoV-2-mediated SG damage. The low level of Hst-5 may lead to prolonged infection and oral inflammation (222). Concurrently, the Th17 response is initiated in the post-infected gland, accompanied by elevated levels of cytokine production including IL-17, IL-22 and IL-23 (222, 223). This finding suggests that the pro-fibrotic factors that contributed to the pathological development of pSS also play a critical role in the post-inflammatory process associated with SARS-CoV-2 infection. In light of these observations, it seems reasonable to hypothesize that long-term oral sequelae in post-COVID patients may be capable of causing secondary SS and triggering SG fibrosis.

COVID-mediated SS and SG fibrosis can be explained by several potential mechanisms (223, 224). Utilizing the aforementioned experimental mouse model with Adv5-cannulated SG, Barone et al. demonstrated the beneficial effect of IL-22 in TLO assembly and autoimmunity (43). IL-22 upregulates the expression of CXCL13 in fibroblasts and CXCL12 in epithelial cells, thus exerting a pro-fibrotic effect (43). Similar to Adv5, it can be postulated that the fibrotic process observed in the SG in the context of the COVID-19 may also be dependent on the presence of IL-22, given the evidence that the Th17 pathway is hyperactivated during the course of infection with the SARS-CoV-2 virus (224). Additionally, approximately 30% of COVID-19 survivors develop COVID-associated pulmonary fibrosis (188, 190, 224). The molecular mechanisms underlying COVID-19-induced pulmonary fibrosis have been elucidated (224). EMT, TGF-β, hyperproliferation of myofibroblasts and high levels of pro-inflammatory IL-2, IL-7, and IFN-γ, are extensively involved in pulmonary fibrosis during SARS-CoV-2 infection (223). Intriguingly, these mechanisms were likewise found in the development of SG fibrosis. These findings may provide novel insight of the pathogenesis of SG fibrosis in COVID patients.

In addition to the effects associated with age, elevated serum titers of rheumatoid factor (RF) and anti-cyclic citrullinated peptide (CCP) have been identified as a significant risk factor for fibrosis in the labial SG, as reported by Katona et al. (225). Follicle dendritic cells contribute to the formation of ectopic germinal centers by promoting B cell differentiation and expansion in a complement-dependent manner (226). Plasmacytoid dendritic cells (pDCs) primarily produce type I interferons and have also been found in ELS (227). pDC cells were closely involved in the SS development due to their IFN-α production (19). The trafficking of circulating pDC cells to the ectopic germinal center of the SG has been documented (15), thereby attracting B cells that home in on the ELS by activating the expression of CXCL13 on macrophages (228). NKp44^+^ NK cells have been linked to focus score and SS severity, although the underlying mechanism was not clear (46). Nonetheless, NKp30^+^ NK cells can interact with SGECs and have pro-inflammatory functions through the secretion of IFN-γ (229).

To date, there is no established diagnostic marker for fibrosis of the SG, which limits the ability to accurately assess its prognosis. In the later stages of the disease, the current therapeutic options for treating fibrosis are limited in their efficacy. Thus, it is imperative to prioritize the prevention of this pathological process requires more attention than treatment. However, there is a possibility that inflamed tissue in SS lesions may not progress into fibrosis during degeneration, even in cases of heavy immune infiltration (230). Kapsogeorgou et al. conducted a 10-year follow-up of SS patients and observed no significant alteration of lesions in their minor salivary glands. The heterogeneity between patients indicates the complexity of the pathogenesis of SS and often results in delays in disease diagnosis (231). This emphasizes the necessity of studying the underlying mechanisms. The acknowledgement of the processes involved in the formation of fibrosis is not yet fully understood, this review may provide valuable insights that will facilitate further exploration of the pathological processes of SS and may provide insights for the development of new therapeutic strategies.
